# EGCG identified as an autophagy inducer for rosacea therapy

**DOI:** 10.3389/fphar.2023.1092473

**Published:** 2023-03-01

**Authors:** Lei Zhou, Yun Zhong, Yaling Wang, Zhili Deng, Yingxue Huang, Qian Wang, Hongfu Xie, Yiya Zhang, Ji Li

**Affiliations:** ^1^ Department of Dermatology, Xiangya Hospital, Central South University, Changsha, China; ^2^ Hunan Key Laboratory of Aging Biology, Xiangya Hospital, Central South University, Changsha, China; ^3^ National Clinical Research Center for Geriatric Disorders, Xiangya Hospital, Central South University, Changsha, China; ^4^ Hunan Binsis Biotechnology Co, Ltd., Changsha, China

**Keywords:** rosacea, EGCG, mTOR, autophagy, skin inflammation

## Abstract

**Background:** Rosacea is a common facial skin inflammatory disease featured by hyperactivation of mTORC1 signaling in the epidermis. Due to unclear pathogenesis, the effective treatment options for rosacea remain limited.

**Methods:** Weighted gene co-expression network analysis (WGCNA) analyzed the relationship between epidermis autophagy and mTOR pathways in rosacea, and further demonstrated it through immunofluorescence and qPCR analysis. A potential therapeutic agent for rosacea was predicted based on the key genes of the WGCNA module. *In vivo* and *in vitro* experiments were conducted to verify its therapeutic role. Drug–target prediction (TargetNet, Swiss, and Tcmsp) and molecular docking offered potential pharmacological targets.

**Results:** WGCNA showed that epidermis autophagy was related to the activation of mTOR pathways in rosacea. Next, autophagy was downregulated in the epidermis of rosacea, which was regulated by mTOR. In addition, the *in vivo* experiment demonstrated that autophagy induction could be an effective treatment strategy for rosacea. Subsequently, based on the key genes of the WGCNA module, epigallocatechin-3-gallate (EGCG) was predicted as a potential therapeutic agent for rosacea. Furthermore, the therapeutic role of EGCG on rosacea was confirmed *in vivo* and *in vitro*. Finally, drug–target prediction and molecular docking revealed that AKT1/MAPK1/MMP9 could be the pharmacological targets of EGCG in rosacea.

**Conclusion:** Collectively, our findings revealed the vital role of autophagy in rosacea and identified that EGCG, as a therapeutic agent for rosacea, attenuated rosacea-like inflammation *via* inducing autophagy in keratinocytes.

## Graphical Abstract

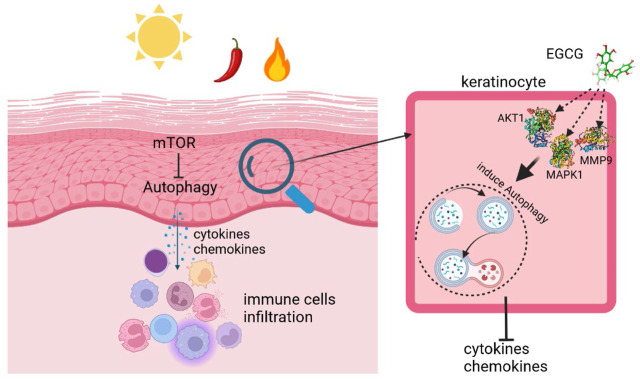

## Background

Rosacea is a common chronic inflammatory skin disorder with a series of features such as facial erythema, telangiectasia, papules, and pustules (Gallo et al., 2018). It significantly impacts the quality of life and affects between 5% and 20% of the population (Gether et al., 2018). The pathogenesis of rosacea is not well understood, but previous studies have shown that the interaction of genetics and a variety of environmental factors may lead to disorders of the skin’s immune system, particularly the abnormal production of cathelicidin LL37, leading to chronic inflammation and abnormal vascular responses of rosacea (Steinhoff et al., 2011; Ahn and Huang, 2018; Awosika and Oussedik, 2018). Due to the ambiguous pathophysiological mechanisms, there is still no effective treatment for rosacea.

The mammalian target of the rapamycin (mTOR) pathway is crucial for various biological processes including cell proliferation, apoptosis, metastasis, and angiogenesis (Deng et al., 2015; Fogel et al., 2015). Our previous work verified hyperactivated mTORC1 signaling in rosacea which promotes rosacea skin inflammation (Deng et al., 2021). Meanwhile, topical administration of rapamycin (mTOR inhibitor) ameliorated clinical lesions in rosacea patients (Deng et al., 2021). However, the underlying mechanism of mTOR signaling in rosacea still needs to be elucidated.

Autophagy is a dynamic process that maintains cellular homeostasis during environmental stress stimuli. Dysregulation of autophagy contributes to the pathogenesis of various skin diseases, including allergic contact dermatitis, atopic dermatitis, and psoriasis (Ohsumi, 2014; Cadwell, 2016). It has been reported that autophagy deficiency led to DNA damage and senescence of keratinocytes (Song et al., 2017). A recent study found that autophagy is essential for the activation of keratinocytes in wound healing (Qiang et al., 2021). In addition, autophagy plays a pivotal role in psoriasiform keratinocyte inflammation (Wang Z. et al., 2021). It is well known that mTOR is an important regulator of the autophagy process (Munson and Ganley, 2015). However, little is known about the link between autophagy and rosacea pathogenesis.

Epigallocatechin-3-gallate (EGCG), a natural polyphenol found in green tea, has many biological activities, including anti-inflammatory, antioxidant, cardioprotective, neuroprotective, and anticancer activities (Nan et al., 2019; Yi et al., 2020; Nan et al., 2021). Studies have revealed EGCG as a potential therapeutic agent for various skin inflammation conditions, including psoriasiform dermatitis (Chamcheu et al., 2018), interface dermatitis (ID) (Braegelmann et al., 2022), and atopic dermatitis (AD) (Noh et al., 2008). Although a clinical trial of four healthy volunteers demonstrated the potential anti-angiogenic effect of EGCG cream (Domingo et al., 2010), whether EGCG has a therapeutic effect on rosacea remains unknown.

Here, we revealed that the autophagy of keratinocytes was associated with the aberrant activation of mTOR signals and contributed to the progression of rosacea. Furthermore, we identified EGCG as a therapeutic agent of rosacea and found that it significantly attenuated rosacea inflammation by inducing autophagy in keratinocytes.

## Methods

### Rosacea transcriptome data

The gene expression array of rosacea (GSE65914) was downloaded from the GEO database. Our previous epidermal transcriptome data (HRA000809) from 18 rosacea tissues and 5 normal skin tissues were downloaded for gene set variation analysis (GSVA).

### GSVA

To investigate the activation of mTOR pathways in rosacea, GSVA was performed using “GSVA” R packages.

### WGCNA

After removing the low-expressed genes (FPKM<1), the genes with the top 25% largest variance were used for WGCNA with power (*β*) = 4 using the “WGCNA” R package as previously described (Li Y. et al., 2021). The genes from modules related to the mTOR pathway with GS > 0.5 were identified as hub genes and used for drug prediction.

### Drug prediction

DGIdb (https://dgidb.org/) was used for drug prediction. The predicted drugs with more than two target genes were collected for further analysis (Cotto et al., 2018; Freshour et al., 2021).

## Animals

For the experiment, 8-week-old female BALB/c mice were purchased from Shanghai SLAC Laboratory Animal Co., LTD. (Shanghai, China). All studies and experimental procedures were approved by the Animal Ethics Committee of Xiangya Hospital of Central South University (No. 201703211). The rosacea-like mouse model was induced as previously described (Agrahari et al., 2020; Kulkarni et al., 2020). Skin inflammation of the mouse model was evaluated by the severity of erythema and edema as previously described (Deng et al., 2021). For EGCG treatment, BALB/c mice were treated with EGCG at a dose of 80 mg/kg per day for seven constitutive days. For topical bafilomycin A1 (BafA1) treatment, mice were injected intradermally with bafilomycin A1 (100 μМ) twice a day for 2 days. The rapamycin treatment was as previously described (Deng et al., 2021).

### Cell culture and treatment

HaCaT cells (Biovector Science Lab, Beijing, China) were cultured according to the manufacturer’s instructions, and the cells were then treated with different doses of EGCG with or without LL-37 (8 μM). For each experiment, 3-MA (10 μM) or BafA1 (10 nM) was added to HaCaT cells 1 h prior to the EGCG treatment. The cells treated with rapamycin, the mTOR inhibitor, were considered a positive control for this study.

### RNA extraction and real-time quantitative PCR (qPCR)

Total RNA was extracted from mouse skin tissue or cells using the Trizol reagent (Invitrogen, United States), and then, cDNA was synthesized using the Maxima H Minus First Strand cDNA Synthesis Kit with dsDNase (Thermo Fisher Scientific, United States). qPCR assay was performed with iTaqTM Universal SYBR^®^ Green Supermix (Bio-Rad, United States) using the CFX Connect Real-Time PCR System (Bio-Rad, United States). qPCR primers are shown in Supplementary Table S1.

### Histological analysis

Skin tissues were fixed overnight with 4% formaldehyde, and sections of 4 μm thickness were used for hematoxylin and eosin (H&E) staining as previously described (Xie et al., 2022). All studies and experimental procedures were approved by the Human Ethics Committee of Xiangya Hospital of the Central South University (No. 201703212).

For immunofluorescence, skin tissues were embedded in OCT and sectioned at 8 μm thickness. The sections were washed with PBS, fixed in 4% frozen paraformaldehyde (PFA) for 15 min, and then blocked for 1 h in PBS containing 1% BSA and 0.3% Triton X-100. Primary antibodies were incubated at 4°C overnight. The sections were washed with PBS and incubated with secondary antibodies for 1 h at room temperature. The nuclei were stained with DAPI. All images were taken using a Zeiss fluorescence microscope and analyzed using Zen2 software (Germany). Anti-LC3 (1:200; Sigma-Aldrich, catalog L7543), anti-CD4 (1:100; eBioscience, catalog 12–0043-82), anti-Beclin1 (1:100; Proteintech, catalog 66665-1-Ig), and Alexa Fluor 488-conjugated goat anti-mouse IgG (H + L) cross-adsorbed secondary Ab (1:500; Invitrogen, catalog A-32723) were used.

### Cell viability assay

Cell proliferation was evaluated using a Cell Counting Kit-8 assay (Vazyme, Nanjing, China). Briefly, 1 × 10^3^ cells/100 μl/well cells were seeded into 96-well plates. The supernatant was removed 48 h later, and 10 μl of the CCK-8 reagent and 100 μl fresh media were introduced per well and incubated for 2 h at 5% CO_2_ and 37°C. Then, the absorbance at 450 nm was measured using the EnSight™ Multimode Plate Reader (PerkinElmer, Waltham, MA).

### Immunoblotting

The skin tissues and cells were lysed in RIPA buffer (Thermo Fisher Scientific, United States). Next, the protein was separated by SDS-PAGE and incubated with primary antibodies, including anti-LC3 (1:1,000; Sigma-Aldrich, catalog L7543), anti-GAPDH (1:5,000; Abcam, catalog ab8245), anti-p62 (1:1,000; Cell Signal Technology, catalog 88,588), anti-S6 (1:1,000; Cell Signal Technology, catalog 2317), and anti-pS6 (Ser240/244) (1:1,000; Cell Signal Technology, catalog 5364).

### Transmission electron microscopy

Cells were treated and collected by trypsinization and fixed in 2.5% glutaraldehyde for 4 h and then refixed in 1% osmium tetroxide for 2 h. After dehydration using a stepwise ethanol series, the cells were embedded in an embedding medium and then polymerized at 60°C for 2 days. The samples were cut on Leica EM UC6 (Leica, Wetzlar, German) at 80 nm thickness and stained with uranyl acetate and lead citrate. Images were acquired using a transmission electron microscope (Hitachi, Tokyo, Japan).

### Ad-mCherry-GFP-LC3 transfection

HaCaT cells were transfected with mCherry-GFP-LC3 adenovirus when they grew to 60%–70% confluence on dishes for 12 h at 37°C. Following treatment with EGCG, LL-37, or BafA1, images were taken using a confocal microscope (Leica, Germany).

### Pharmacological targets of EGCG

We used accessible online tools to predict the potential pharmacological targets of EGCG, including TargetNet, Swiss, and TCMSP (Wishart et al., 2018). Then, the candidate targets were identified using the UniProt database (Li R. et al., 2021).

### Molecular docking

The PubChem database (https://pubchem.ncbi.nlm.nih.gov/) was used to obtain the molecular structure of EGCG (CID-65064). The PDB database (https://www.rcsb.org/) was used for the protein structures of AKT1 (6HHG), MAPK1 (6DCG), and MMP9 (6ESM). Maestro software was used for molecular docking (Zhang H. et al., 2021).

### Statistical analysis

Statistical analysis was conducted with GraphPad Prism (8.0.0) (San Diego, California United States). All data were displayed as the mean ± SEM of three independent experiments. Unpaired Student’s t-test was used for the comparison of two groups, and one-way ANOVA followed by Dunnett’s test was used for multiple comparisons. The level of statistical significance was set at *p* < 0.05.

## Results

### WGCNA identified the keratinocyte autophagy associated with the mTOR pathway in rosacea

**FIGURE 1:**
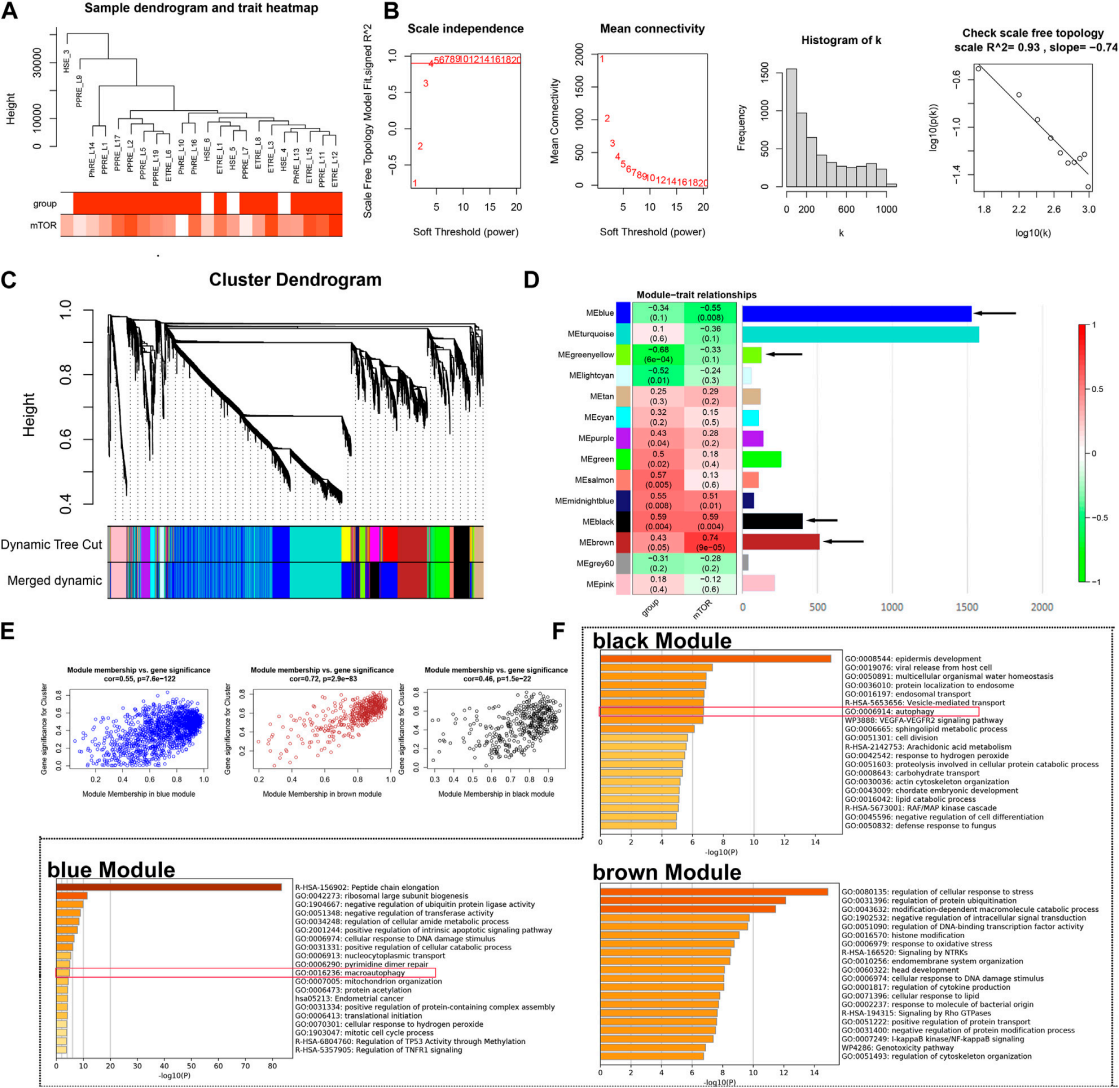
WGCNA. **(A)** Sample cluster analysis associated with clinical characters. **(B)** Scale-free fitting index analysis and the mean connectivity for various soft-threshold powers. **(C)** Gene dendrogram and module colors of WGCNA. **(D)** Correlation analysis between modules and clinical characters. **(E)** Relationship between GS and MM in the blue, brown, and black modules. **(F)** GO analysis of genes from blue, brown, and black modules, respectively.

Our previous study identified the important role of the mTOR pathway in rosacea; however, the potential mechanism remains unknown (Deng et al., 2021). Here, based on our previous epidermis transcriptome data, GSVA identified the activation of the mTOR pathway in rosacea (Supplementary Figure S1). Next, we used WGCNA to identify the rosacea-related and mTOR pathway-related genes in the epidermis of rosacea. A total of 5,278 genes were used for WGCNA, and one abnormality (HSE_3) was removed (Figure 1A). The soft threshold *β* = 4 and scale-free *R* ^2^ = 0.93 are shown in Figure 1B. After merging the similar modules, 14 modules were obtained as shown in Figure 1C. The relationships between the mTOR pathway and modules are shown in Figure 1D. The black module (r = 0.59, *p* = 0.004) and brown module (r = 0.74, P = 9e-5) were positively associated with the mTOR pathway, while the blue module (r = -0.55, *p* = 0.008) was negatively associated with the mTOR pathway. The relationship between GS and MM in black, brown, and blue modules is shown in Figure 1E. The GO enrichment analysis demonstrated that the genes in the blue and black modules were enriched in the autophagy-related signal pathways using Metascape (http://metascape.org/) (Figure 1F). These results indicated that the activated mTOR pathway could affect keratinocytes’ autophagy in rosacea.

### Autophagy was reduced in the keratinocytes and aggravated rosacea-like inflammation

**FIGURE 2:**
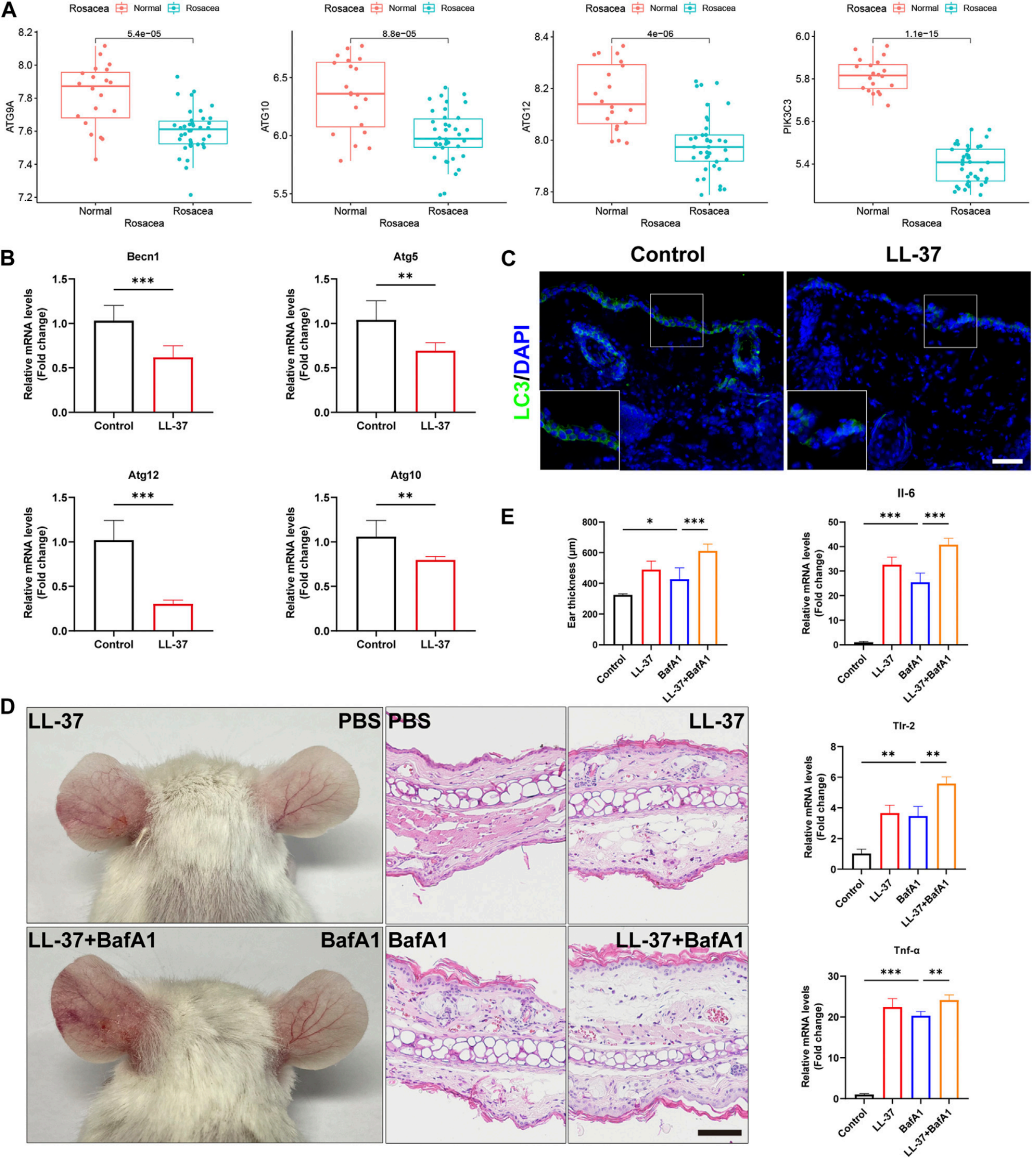
Autophagy was reduced in the keratinocytes and aggravated rosacea-like inflammation. **(A)** Expression of the autophagy markers, ATG9A, ATG10, ATG12, and PIK3C3, in the epidermis of rosacea patients and normal subjects. **(B)** mRNA expression levels of Becn1, Atg5, Atg10, and Atg12 in LL-37-induced mouse skin lesions. **(C)** Immunofluorescence analysis of LC3 in skin lesions from control mice and LL37-induced mice. Scale bar: 50 μm. **(D)** Representative images and HE straining of mice injected with BafA1 and/or LL-37 showing erythema on the ear. **(E)** Measurement of the mouse ear thickness. The mRNA expression levels of Il6, Tlr-2, and Tnf-α. (n = 5 for each group). All results are representative of at least three independent experiments. Data represent the mean ± SEM. One-way ANOVA with Bonferroni’s *post hoc* test was used for statistical analyses. *p < 0.05, **p < 0.01, and ***p < 0.001.

To determine the roles of autophagy in rosacea, we analyzed the expression levels of the autophagy-related markers in rosacea lesion tissues and normal skin tissues. As shown in Figure 2A and Supplementary Figure S2, the expression of autophagy-related genes (ATG9A, ATG10, ATG12, and PIK3C3) was evidently decreased in rosacea lesions compared with normal skin tissues in the GSE65914 dataset. Immunofluorescence revealed decreased becline1 in the lesioned skin of rosacea patients (Supplementary Figure S3). These results were confirmed in LL-37-induced rosacea-like mouse models. We observed that the mRNA expressions of autophagy-related genes (Becn1, Atg5, Atg10, and Atg12) were decreased in LL-37-induced mouse skin tissue (Figure 2B). The immunofluorescence analysis also revealed that the LC3 expression was much lower in the epidermis of LL-37-induced rosacea-like lesioned skin than in control mouse skin tissues (Figure 2C).

Next, we investigated whether autophagy affects LL-37-induced rosacea-like inflammation. For that, 8-week-old BALB/c female mice were injected intradermally with LL-37 alone, bafilomycin A1 (autophagy inhibitor) alone, or co-injected with both LL-37 and bafilomycin A1. Enhanced ear redness and thickness were observed, accompanied by an increase in Il-6, Tlr-2, and Tnf-α (Figures 2D, E). We also found that Cxcl1, Cxcl15, Cd68, Itgam, Cma1, and Tpsab1 were increased when treated with BafA1 alone or with LL-37 + BafA1 (Supplementary Figure S4). In addition, in our previous studies, we observed that rapamycin, an agonist of autophagy, prevents the development of rosacea-like skin inflammation (Deng et al., 2021). In the present study, we found that the mRNA expression of autophagy-related genes Becn1, Atg5, Atg10, and Atg12 was significantly increased in LL37-induced rosacea lesions after topical rapamycin treatment (Supplementary Figure S5). Altogether, these results demonstrated that autophagy was reduced in keratinocytes of rosacea, and autophagy impairment/improvement aggravated/ameliorated rosacea-like skin inflammation.

### EGCG was identified as a candidate drug for rosacea

**FIGURE 3:**
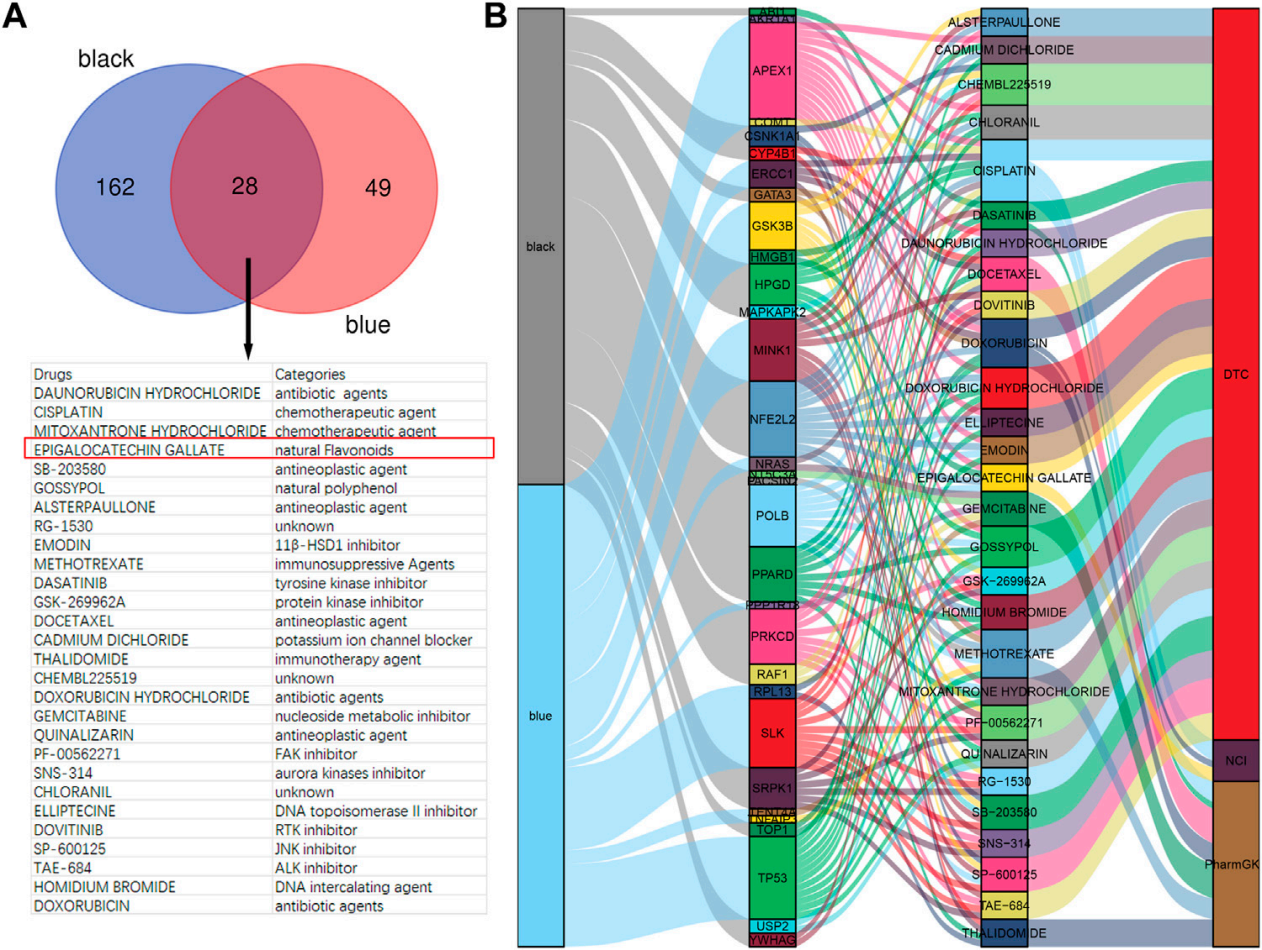
EGCG is a candidate drug for rosacea. **(A)** Overlapped drugs predicted by DGIdb. **(B)** Sankey diagram revealed the relationship between modules, hub genes, and drugs.

To investigate the candidate drugs for rosacea, the hub genes from black and blue modules were input into DGIdb. In total, 190 drugs targeting 23 genes from the black module and 77 drugs targeting 15 genes from the blue module were identified, and 28 drugs overlapped (Figure 3A). The Sankey diagram revealed the detailed relationship between hub genes and 28 drugs (Figure 3B). Among them, EGCG has been reported to present anti-inflammatory and immunoregulatory effects and has been increasingly recognized worldwide for its low cost, easy-to-obtain nature, low toxicity, low side effects, and high tolerance. So, EGCG was selected for further study.

### EGCG attenuated LL-37-induced rosacea-like dermatitis

**FIGURE 4:**
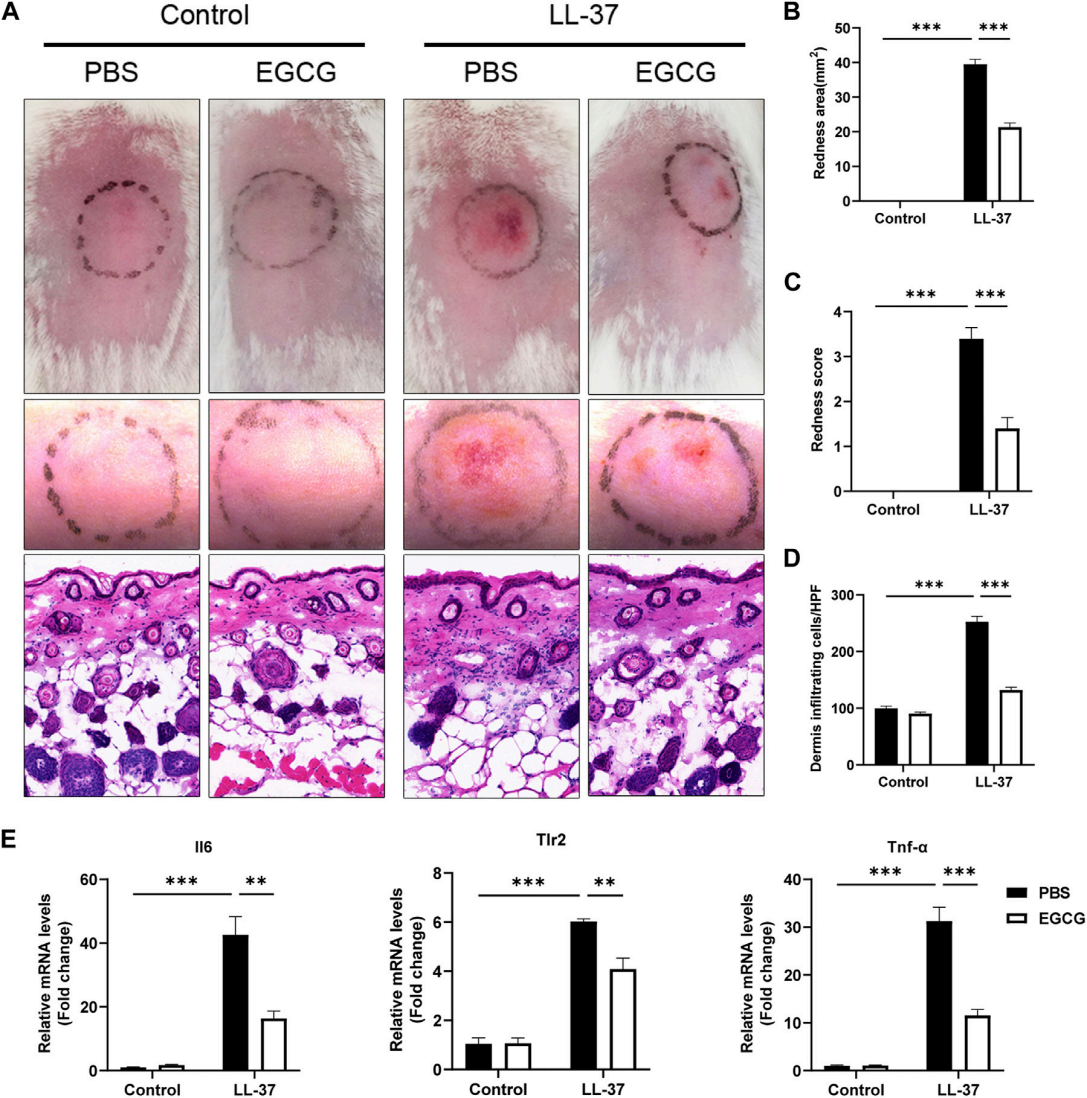
Effect of EGCG on LL37-induced rosacea-like mice. **(A)** Skin manifestation of different groups. Images were taken 48 h after the first LL37 administration. Scale bar: 50 μm. The severity of inflammatory responses on the skin was assessed in the redness area **(B)**, redness score **(C)**, and quantitative result of HE staining for dermal cellular infiltrates **(D)**. **(E)** mRNA expression levels of Il6, Tlr2, and TNF-α in skin lesions (n = 5 for each group). All results are representative of at least three independent experiments. Data represent the mean ± SEM. One-way ANOVA with Bonferroni’s *post hoc* test was used for statistical analyses. **p < 0.01 and ***p < 0.001.

We initially investigated the potential therapeutic effect of EGCG on rosacea in an LL-37-induced mouse model. As shown in Figure 4A, EGCG treatment significantly ameliorated the LL37-induced rosacea-like lesions. The average redness area and score were dramatically reduced in the EGCG group compared with the PBS group (Figures 4B, C). Histological analysis showed that treatment with EGCG resulted in the reduction of immune infiltration in the dermis (Figures 4A–D). Meanwhile, EGCG treatment also reduced the expression of pro-inflammatory cytokines, including Il-6, Tlr-2, and Tnf-α in LL-37-induced rosacea-like lesions (Figure 4E). Moreover, EGCG also reduced the expressions of the neutrophil-attracting chemokines (Cxcl15 and Cxcl1), macrophage markers (Cd68 and Itgam), and mast cell-related genes (Tpsab1 and Cma1) in LL-37-induced rosacea-like lesions (Supplementary Figure S6A). The infiltration of CD4^+^ T cells and the expression of Stat1, Stat3, and IL-17A were repressed by EGCG treatment in rosacea-like mice (Supplementary Figures S6B–D). These results demonstrated the therapeutic effect of EGCG in rosacea-like dermatitis in mice.

### EGCG decreased LL-37-induced inflammation in keratinocytes

**FIGURE 5:**
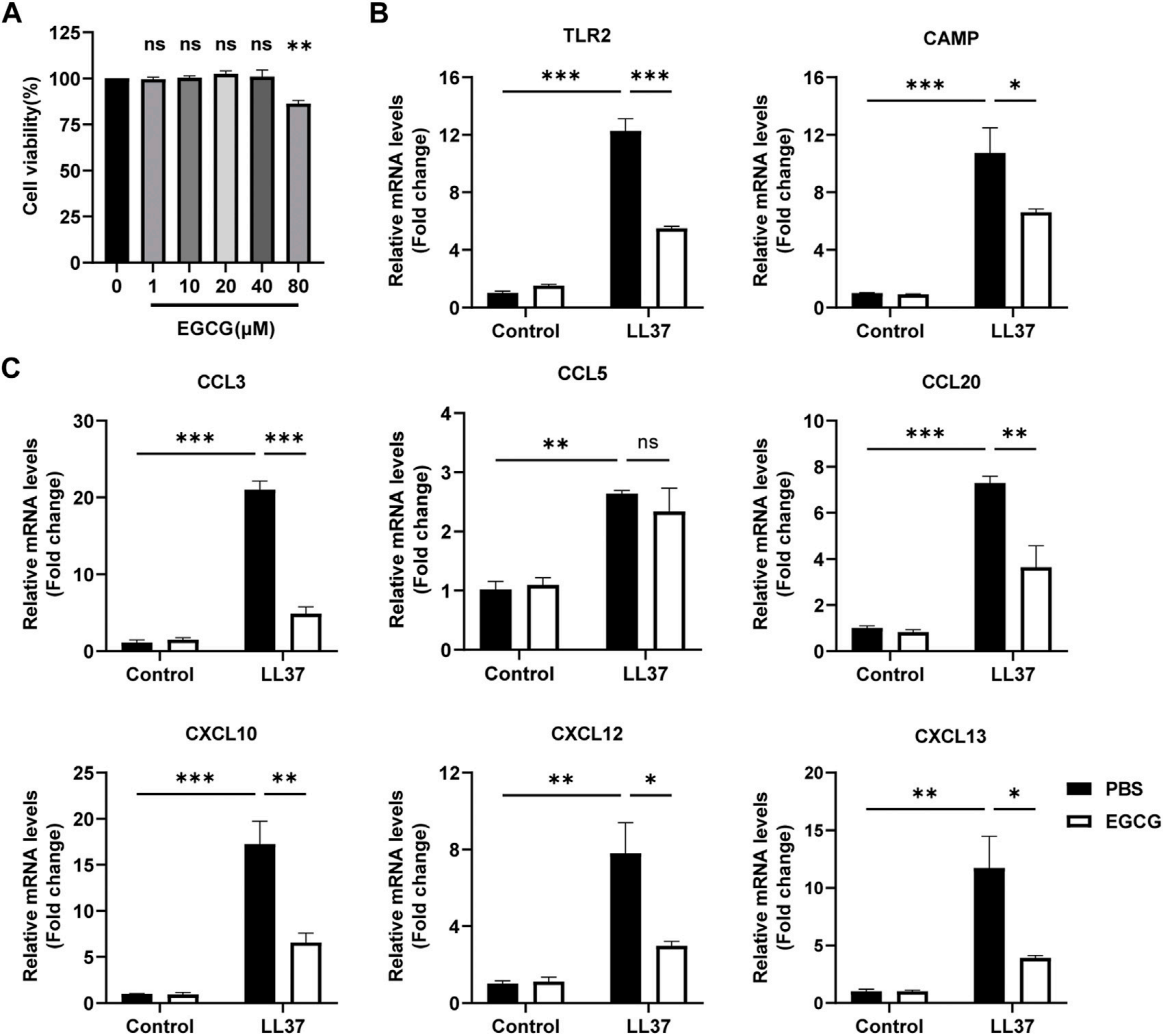
EGCG decreased the production of cytokines and chemokines related to rosacea in keratinocytes. **(A)** Effect of different concentrations of EGCG on cell viability is determined by CCK-8 assay. **(B)** mRNA expression levels of TLR2 and CAMP. **(C)** mRNA expression levels of CCL3, CCL5, CCL20, CXCL10, CXCL12, and CXCL13. All results are representative of at least three independent experiments. Data represent the mean ± SEM. One-way ANOVA with Bonferroni’s *post hoc* test was used for statistical analyses. *p < 0.05, **p < 0.01, and ***p < 0.001. ns, no significance.

First, we detected the role of EGCG on keratinocytes *in vitro*. We found that the concentrations of 80 μM EGCG repressed the viability of HaCaT cells, and drug concentrations of 10, 20, and 40 μM were chosen in the following cell experiments (Figure 5A). Next, we demonstrated that EGCG treatment reduced LL37-induced TLR-2 and CAMP, the key rosacea markers (Yamasaki et al., 2007; Yamasaki et al., 2011; Zhang J. et al., 2021), and expression in the HaCaT cells (Figure 5B). Considering the pivotal role of keratinocytes in producing excessive pro-inflammatory cytokines and chemokines in the pathogenesis of rosacea (Steinhoff et al., 2011), we demonstrated the inhibitory effects of EGCG on cytokine and chemokine expression in HaCaT cells. The expressions of pro-inflammatory cytokines and chemokines, including CXCL10, CCL20, CCL3, CCL5, CXCL12, and CXCL13, were analyzed using the qPCR assay. All these genes except CCL5 were significantly reduced by EGCG treatment (Figure 5C). Thus, we concluded that EGCG repressed LL-37-induced keratinocyte inflammation.

### EGCG reduced rosacea-like inflammation by inducing keratinocyte autophagy

**FIGURE 6:**
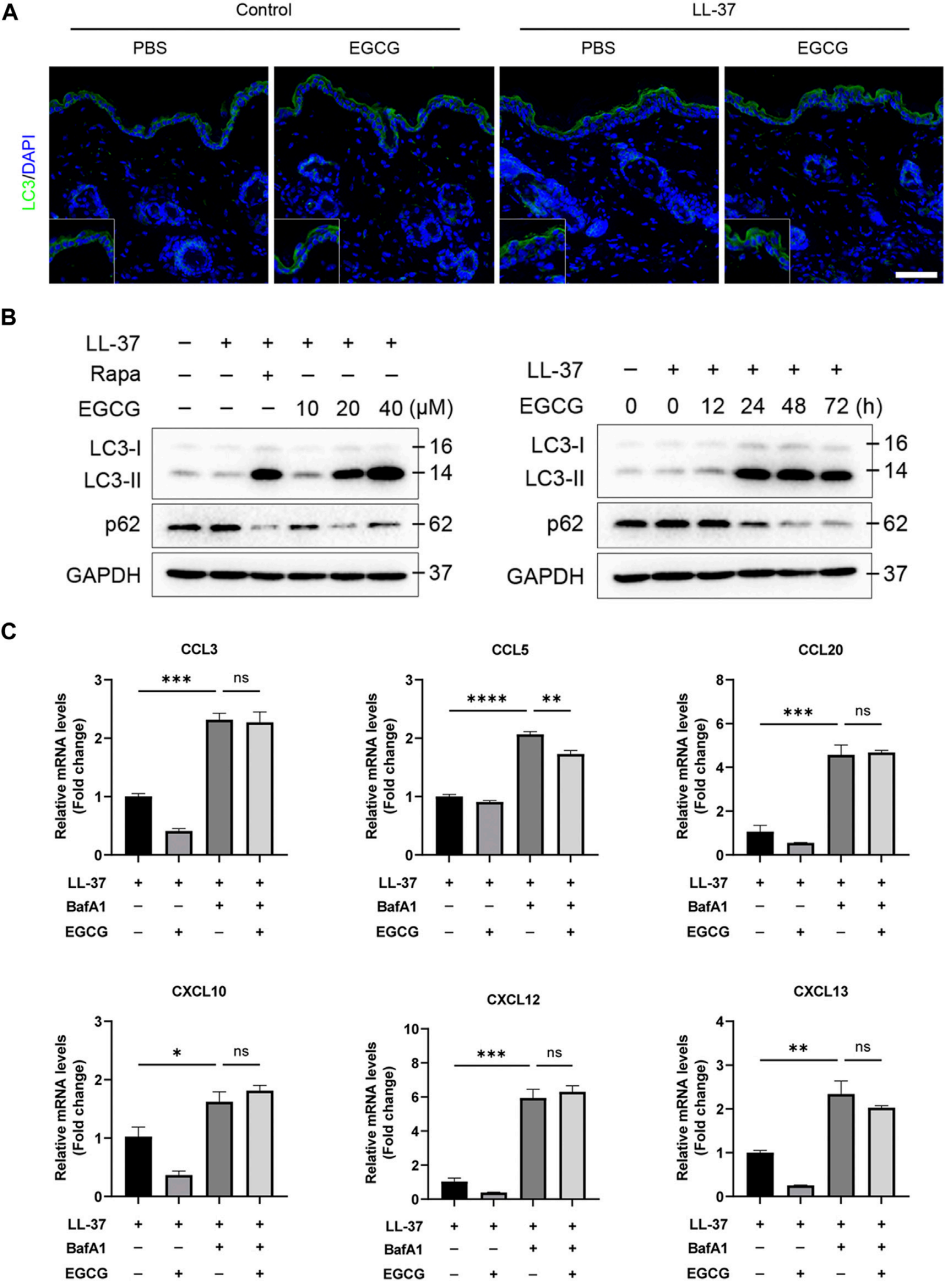
EGCG reduced rosacea-like inflammation by inducing keratinocyte autophagy. **(A)** LC3 immunofluorescence staining (green) in LL-37-induced mice treated with or without EGCG. DAPI staining (blue) indicates nuclear localization. Scale bar: 50 μm. **(B)** Representative immunoblot analysis for the expression of LC3 and p62 in a dose- and time-dependent manner of EGCG treatment. **(C)** Inhibition of autophagy impairs the anti-inflammatory role of EGCG in HaCaT cells. The mRNA expression levels of CCL3, CCL5, CCL20, CXCL10, CXCL12, and CXCL13. All results are representative of at least three independent experiments. Data represent the mean ± SEM. One-way ANOVA with Bonferroni’s *post hoc* test was used for statistical analyses. *p < 0.05, **p < 0.01, and ***p < 0.001. ns, no significance.

It has been reported that autophagy effectively protects keratinocytes against injury in inflammatory skin diseases (Hou et al., 2020; Kim et al., 2021). To confirm whether the anti-inflammatory effect of EGCG could be due to the induction of autophagy in rosacea, we detected the autophagy levels in rosacea-like mice after EGCG treatment. Here, we found that EGCG could induce keratinocyte autophagy in LL37-induced rosacea-like mice (Figure 6A). Next, we detected the role of EGCG in autophagy in LL37-treated HaCaT cells. The HaCaT cells were treated with 10, 20, and 40 μM EGCG or rapamycin (autophagy agonist) in the presence of LL-37, and subsequent autophagy events were monitored by western blotting. As shown in Figures 6A, B, LC3-I gradually transformed into LC3-II with the increase in EGCG concentration and treatment time. To determine the role of EGCG-induced autophagy in LL37-induced keratinocyte inflammation, BafA1, an autophagy inhibitor, was included in the ensuing studies. qPCR analysis showed that EGCG-repressed pro-inflammatory cytokine and chemokine expression, including CCL3, CCL5, CCL20, CXCL10, CXCL12, and CXCL15, was reversed by BafA1 treatment (Figure 6C).

**FIGURE 7:**
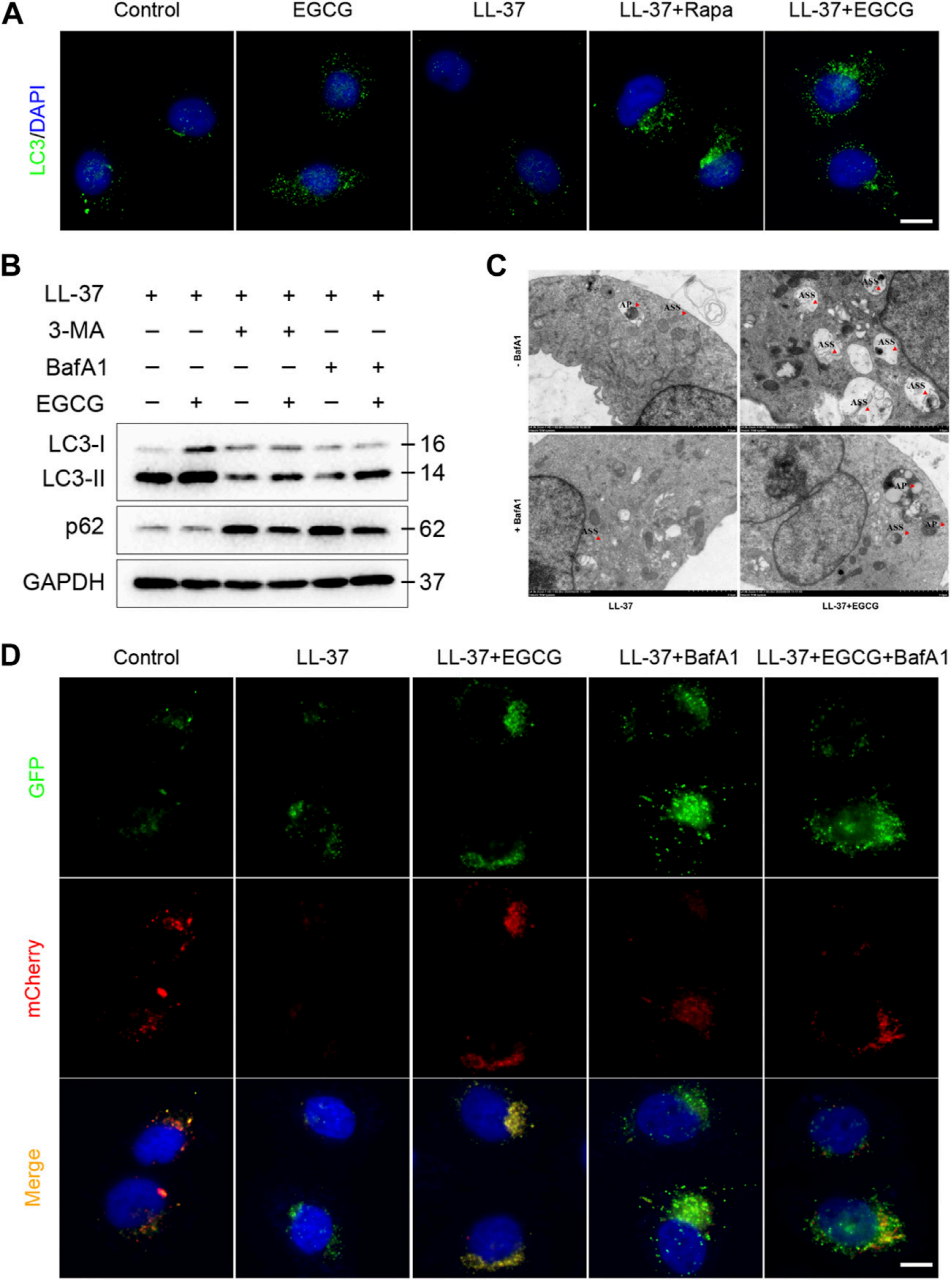
EGCG-induced autophagy in LL-37-induced HaCaT cells. **(A)** Immunostaining of LC3 in HaCaT keratinocytes treated with LL37 and/or EGCG for 24 h. DAPI staining (blue) indicates nuclear localization. Scale bar: 20 μm. **(B)** Representative immunoblot analysis of autophagy marker proteins in response to various treatments. **(C)** Representative TEM images showing the ultrastructure of HaCaT cells incubated with EGCG with or without BafA1 in the presence of LL-37. The red arrowheads indicate the autophagic vacuoles, respectively. AP, autophagosome; ASS, autolysosome. **(D)** HaCaT cells were transfected with the mCherry-GFP-LC3 plasmid and then treated with EGCG and/or BafA1 in the presence of LL-37 for 24 h. Nuclei were stained with DAPI. Scale bar: 20 μm. All results are representative of at least three independent experiments.

Meanwhile, to examine whether the mechanism of EGCG on autophagy was due to an increase in the autophagy level and not due to the blocking of autophagy flux, we further analyzed p62 protein expression, which reflects the level of autophagosome clearance and negatively correlates with autophagy (Lamark et al., 2017). After EGCG treatment, there was a significant decrease in the p62 expression level (Figure 6B). EGCG significantly reduced p62 expression but induced LC3-II levels in a dose- and time-dependent manner. Cytoplasmic LC3 puncta formation is the hallmark event of autophagy (Schaaf et al., 2016). Thus, we examined EGCG induction of LC3 puncta formation after treating the HaCaT cells with EGCG by immunofluorescent staining. We observed that LC3 puncta formation was considerably augmented in the EGCG treatment group, while it was reduced in the LL-37-induced HaCaT cells compared with the untreated vehicle control (Figure 7A).

Furthermore, to clarify the correlation between EGCG and autophagy induction, 3-MA and bafilomycin A1 (BafA1), autophagy inhibitors, were employed in the subsequent studies. Immunoblot analysis showed that 3-MA and BafA1 blocked the EGCG-induced conversion of LC3-I to LC3-II, while p62 degradation induced by EGCG was impeded by autophagy inhibitors in LL-37-induced conditions (Figure 7B). Likewise, we found that the HaCaT cells co-treated with EGCG and LL-37 showed abundant autophagolysosomes under transmission electron microscopy. However, in contrast, the cells treated with merely LL-37 or BafA1 showed a limited number of autophagosomes and autophagolysosomes (Figure 7C). Next, tandem mCherry-GFP-LC3 fluorescence microscopy assay and transmission electron microscopy were performed to assess autophagosome and autophagolysosome formation. Our results indicated that the number of autophagosomes (green spots) and autolysosomes (yellow spots) in the EGCG treatment group was significantly increased compared to other groups, which suggested that EGCG enhanced autophagy flux in LL-37-induced HaCaT cells (Figure 7D).

Taken together, these data strongly suggested that EGCG attenuated LL-37-induced inflammation by increasing autophagy induction and autophagy flux in keratinocytes.

### ATK1, MAPK1, and MMP9 could be direct targets of EGCG in rosacea

**FIGURE 8:**
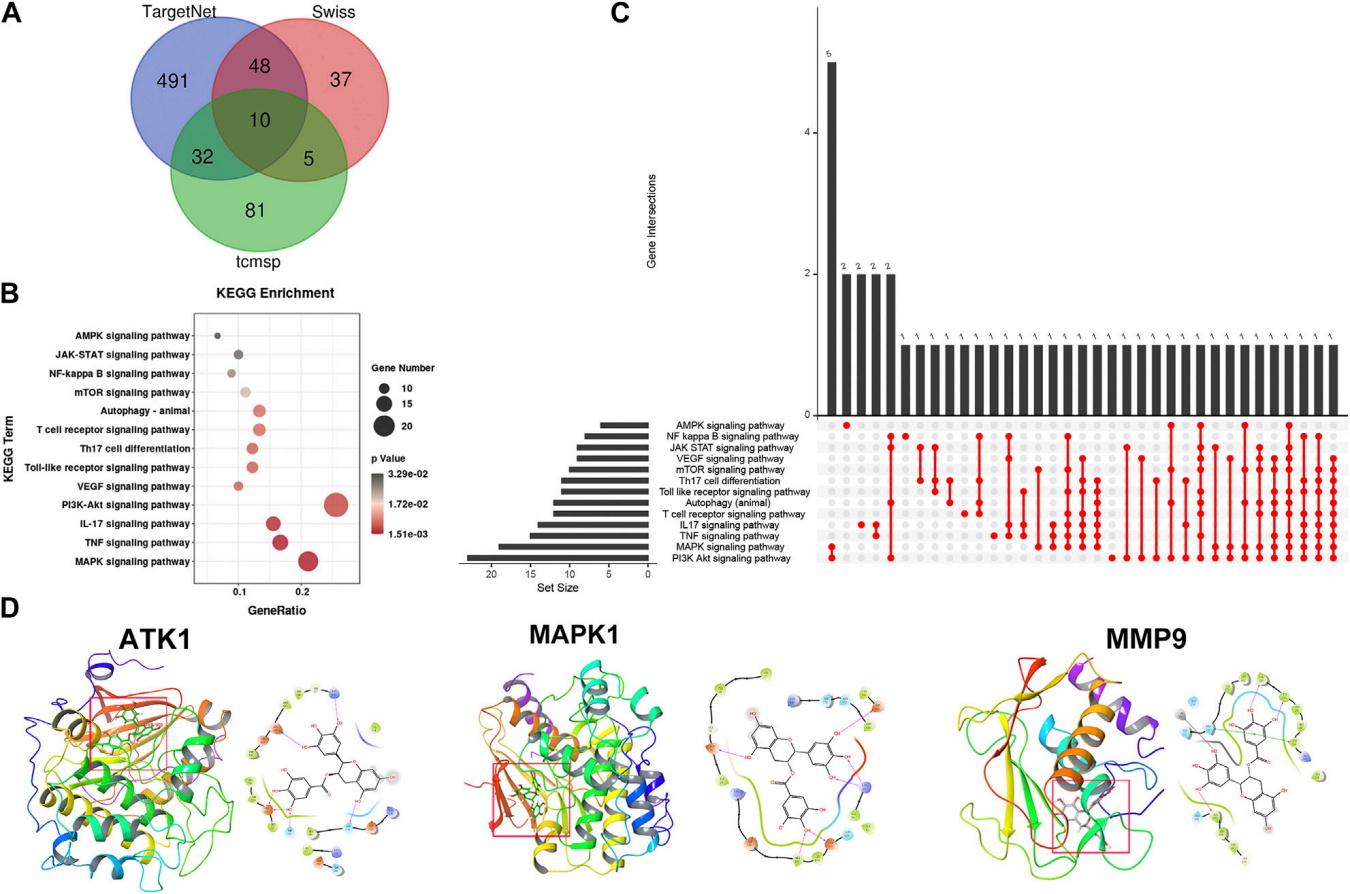
Pharmacological targets of EGCG in rosacea. **(A)** Overlapped target genes of EGCG in TargetNet, Swiss, and Tcmsp. **(B)** KEGG enrichment analysis of EGCG-targeted signaling pathways. **(C)** Upset diagram of EGCG-targeted signaling pathways. **(D)** Molecular docking revealed the binding targets of EGCG.

To explore the specific molecular mechanism of EGCG-induced autophagy, three databases (TargetNet, Swiss, and Tcmsp) were used to predict the target of EGCG in rosacea, and 95 target genes were predicted in two or more databases at the same time (Figure 8A). The GO analysis revealed the enrichment of these target genes in rosacea-related, autophagy-related, and mTOR-related pathways (Figures 8B, C). Among them, ATK1, MAPK1, and MMP9 were the key molecules in these pathways. Subsequent molecular docking was used to predict the binding of EGCG to ATK1, MAPK1, and MMP9 (Figure 8D). ATK/MAPK pathways were reported as a regulator of autophagy (Yuan et al., 2022). So, we speculated that EGCG could regulate autophagy by targeting ATK1, MAPK1, and MMP9 in rosacea.

## Discussion

Although significant effort is devoted to revealing pathogenesis and developing new therapeutic agents, the current therapeutic strategies for rosacea are still unsatisfactory (Logger et al., 2020; Wang B. et al., 2021; Kim et al., 2022). In this study, we revealed the important role of epidermis autophagy in rosacea and demonstrated EGCG as an effective agent for rosacea treatment, which attenuated rosacea-like inflammation *via* inducing keratinocyte autophagy.

The mTOR pathway is a crucial signal transduction pathway implicated in various physiological and pathological processes (Deng et al., 2015; Fogel et al., 2015). Our previous work demonstrated the important role of hyperactivated mTOR signaling in rosacea (Deng et al., 2021). In this study, an upregulated mTOR pathway in the epidermis of rosacea patients was confirmed using GSVA. Subsequently, WGCNA revealed the potential regulation of mTOR signaling on autophagy in the epidermis of rosacea. Autophagy is essential for the homeostasis of keratinocytes, and dysregulation of autophagy contributes to the pathogenesis of skin diseases and has been shown to play a critical role in inflammatory skin disorders, including atopic dermatitis, psoriasis, and allergic contact dermatitis (Cadwell, 2016). In this study, we found that autophagy was decreased and contributed to the progression of rosacea. mTOR is a well-known regulator of autophagy (Munson and Ganley, 2015). It has been shown that IL-17A-activated PI3K/AKT/mTOR signaling contributed to the inflammatory response of psoriasis partly by inhibiting autophagy in keratinocytes (Varshney and Saini, 2018). Rapamycin, a well-known mTOR inhibitor, alleviated psoriasis-like dermatitis by inducing autophagy (Kim et al., 2021). Our previous study revealed the therapeutic role of rapamycin in rosacea. We also revealed the induction of autophagy by rapamycin in rosacea-like dermatitis, implying that autophagy was a novel therapeutic target for rosacea.

In recent years, natural medicinal products and plant extracts have been highly sought after for therapeutic drugs with the advantages of cost effectiveness, high bioactivity, abundant content, and safety. EGCG, a natural polyphenol found in green tea, has been reported to have many biological activities, including anti-inflammatory, antioxidant, cardioprotective, neuroprotective, and anticancer activities (Nan et al., 2019; Huang et al., 2020; Yi et al., 2020). In this study, based on the mTOR signal and autophagy-related genes, EGCG was predicted as a candidate drug for rosacea. The *in vivo* and *in vitro* experiments showed that EGCG attenuated rosacea-like inflammation by inducing keratinocyte autophagy. Consistent with our results, EGCG was proven therapeutic to various diseases by inducing cytoprotective autophagy (Wu et al., 2021). Cytoplasmic LC3 puncta formation is the hallmark of autophagy (Schaaf et al., 2016); the blocking of autophagy flux and an increase in the autophagy level lead to increased LC3-II (Lamark et al., 2017). In the present study, it was observed that EGCG promoted the formation of autophagosomes and autophagolysosomes accompanied in a dose- and time-dependent manner. Subsequently, the target prediction and molecular docking showed that ATK1, MAPK1, and MMP9 were the potential targets of EGCG. ATK/MAPK pathways were reported as a regulator of autophagy (Yuan et al., 2022).

## Conclusion

In summary, we demonstrated a contribution of impaired autophagy in rosacea pathogenesis and implied EGCG as an effective treatment strategy for rosacea, which attenuated rosacea-like inflammation *via* inducting autophagy in keratinocytes.

## Data Availability

The original contributions presented in the study are included in the article/Supplementary Material; further inquiries can be directed to the corresponding authors.

## Abbreviations

EGCG, epigallocatechin-3-gallate; mTOR, mammalian target of rapamycin; ID, interface dermatitis; AD, atopic dermatitis; GSVA, gene set variation analysis; PFA, paraformaldehyde frozen.
