# Supplementary material for: EGCG identified as an autophagy inducer for rosacea therapy
Source: Front Pharmacol. 2023 Mar 1;14:1092473. doi: 10.3389/fphar.2023.1092473 (PMC10014537; doi:10.3389/fphar.2023.1092473)
Supplement: Supplementary file 1 [file Table1.DOCX]

###
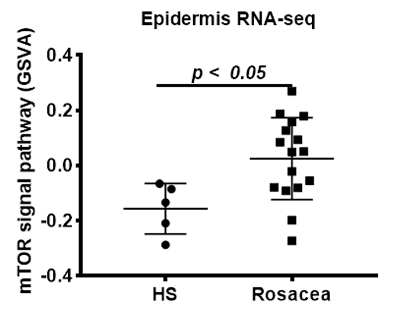
Figure S1. The GSVA analysis revealed the activation of the mTOR signaling pathway in the rosacea.


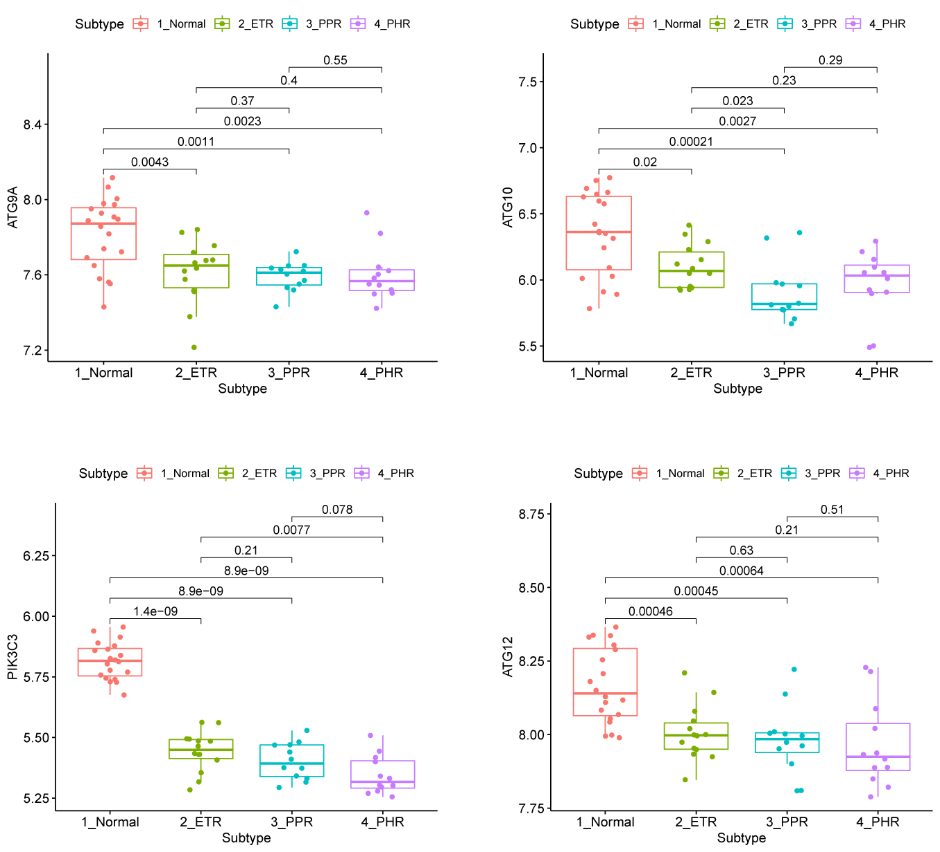
Figure S2. Expression of the autophagy marker, ATG9A, ATG10, PIK3C3, and ATG12 in the different rosacea subtypes using GSE65914 dataset.


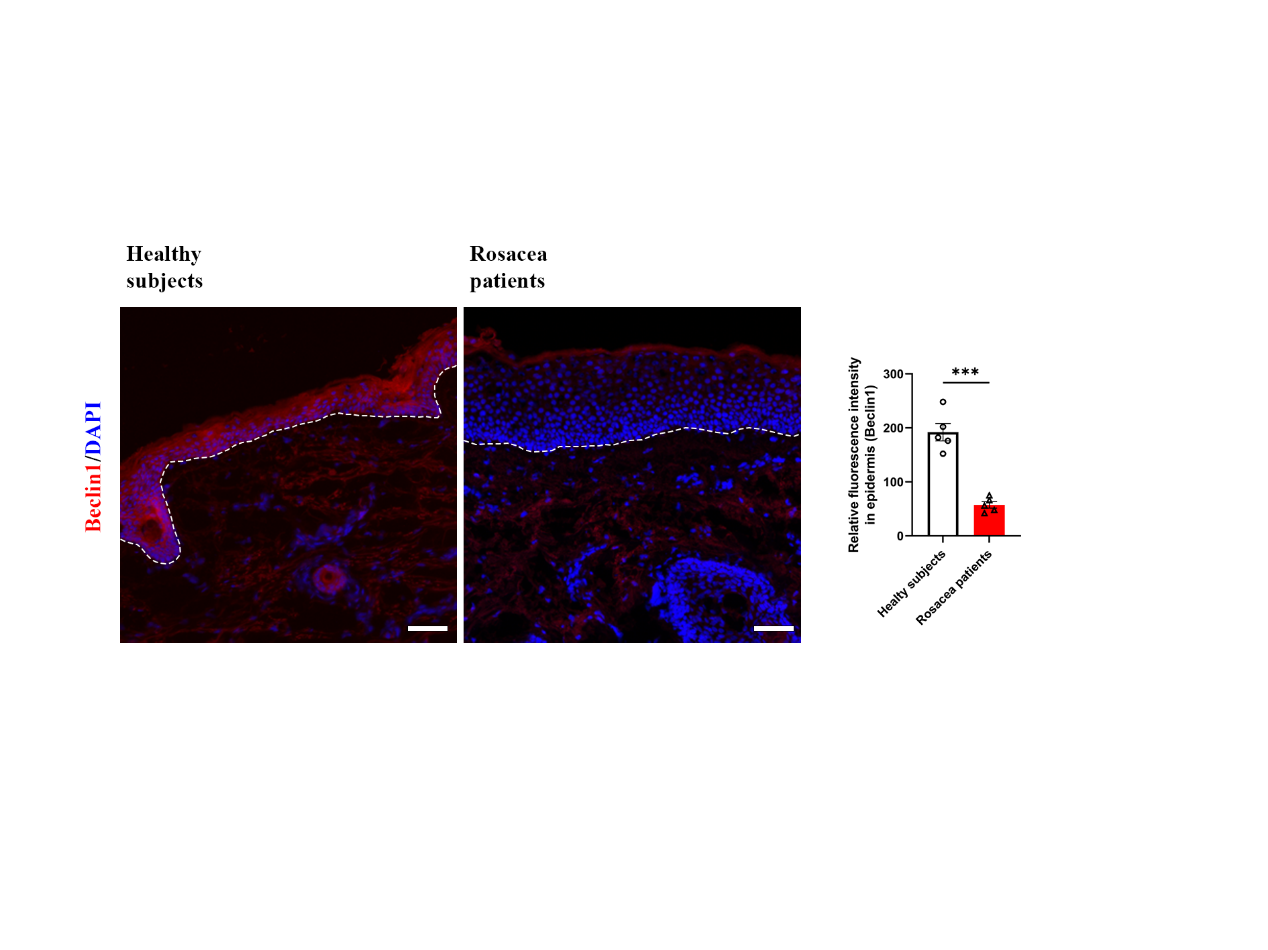
 Figure S3. The expression of becline1 in lesional skin of rosacea patients and normal facial skin of healthy subjects.


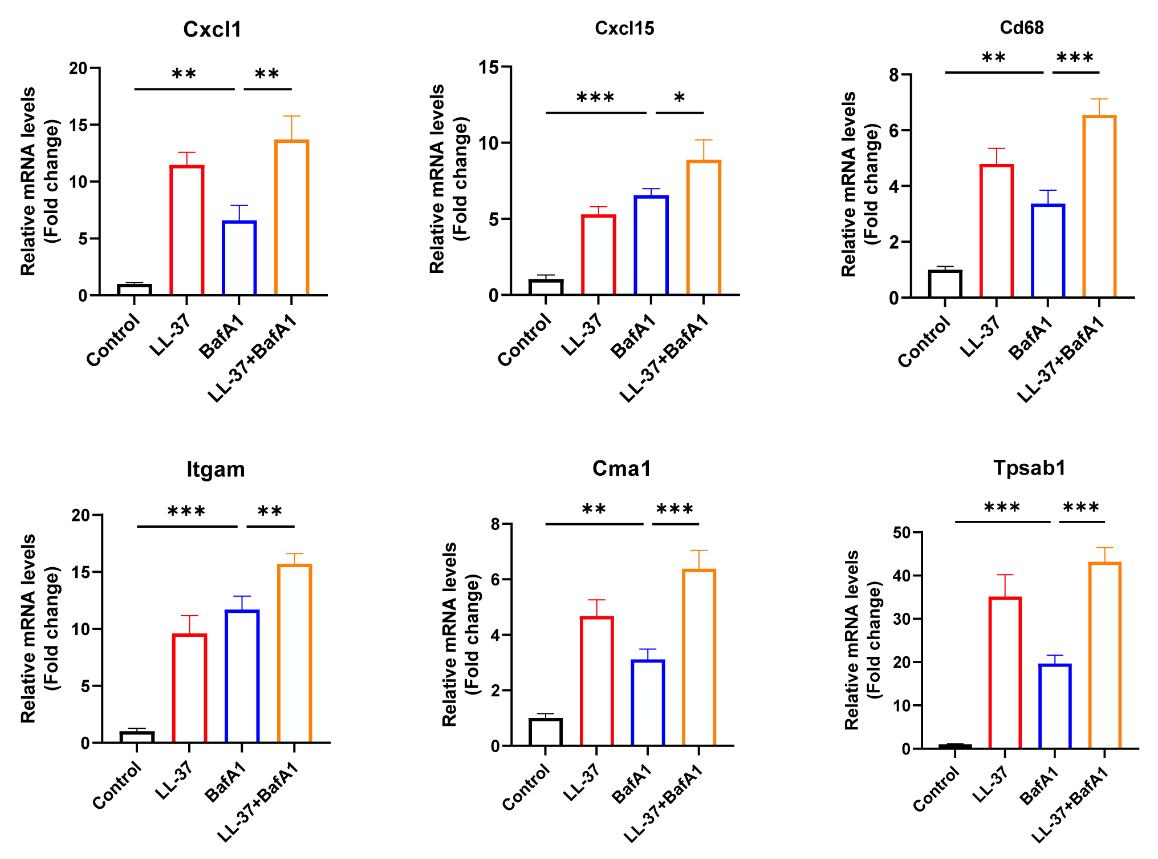


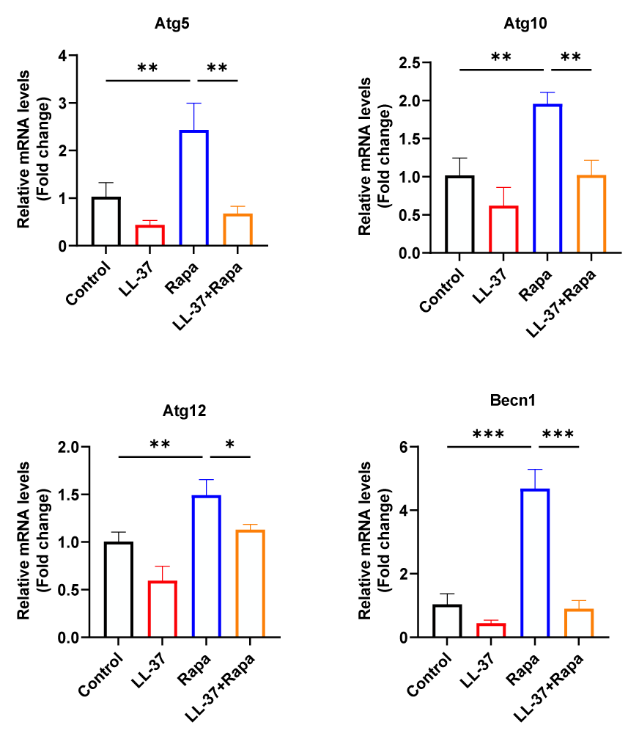
Figure S4. The mRNA expression levels of Cxcl1, Cxcl15, Cd68, Itgam, Cma1, and Tpsab1. (n = 5 for each group). All results are representative of at least 3 independent experiments. Data represent the mean ± SEM. One-way ANOVA with Bonferroni’s post hoc test were used for statistical analyses. *P < 0.05, **P < 0.01, ***P < 0.001.


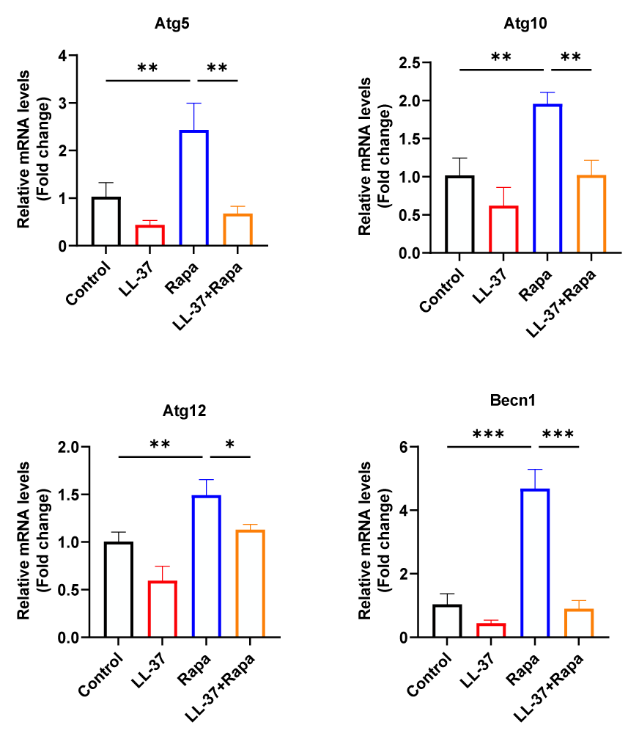


Figure S5. Inhibition of mTOR induces autophagy in LL-37-induced mice. The mRNA expression levels of Atg5, Atg10, Atg12, and Becn1. All results are representative of at least 3 independent experiments. Data represent the mean ± SEM. One-way ANOVA with Bonferroni’s post hoc test were used for statistical analyses. *P < 0.05, **P < 0.01, ***P < 0.001.


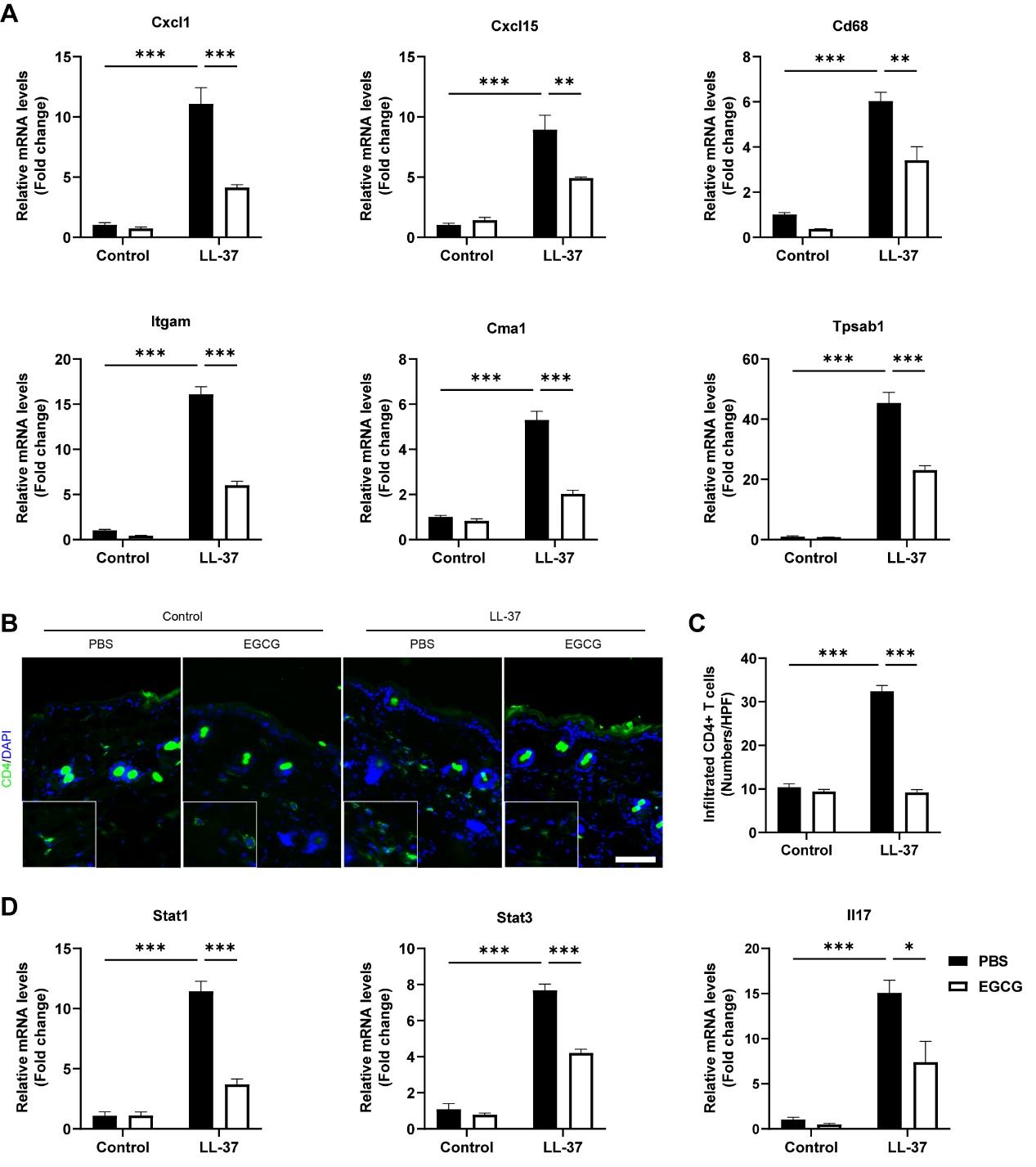


**Figure S6.** Effect of EGCG on immune response in LL37-treated skin. (A) The mRNA expression levels of Cxcl1, Cxcl15, Cd68, Itgam, Cma1, and Tpsab1. (B) Immunofluorescence analysis of CD4 in skin lesions from control mice and LL37-induced mice pretreated with vehicle or EGCG. Scale bar: 50 μm. (C) The infiltration of CD4 positive cells was quantified. (D) The mRNA expression levels of Stat1, Stat3, and Il17. All results are representative of at least 3 independent experiments. Data represent the mean ± SEM. One-way ANOVA with Bonferroni’s post hoc test were used for statistical analyses. *P < 0.05, **P < 0.01, ***P < 0.001.

## Appendix Table S1. List of qPCR primers in this study.

| Human-GAPDH-F | GGAGCGAGATCCCTCCAAAAT |
| --- | --- |
| Human-GAPDH-R | GGCTGTTGTCATACTTCTCATGG |
| Human-TLR2-F | ATCCTCCAATCAGGCTTCTCT |
| Human-TLR2-R | GGACAGGTCAAGGCTTTTTACA |
| Human-CAMP-F | AGGTCCTCAGCTACAAGGAAG |
| Human-CAMP-R | TCTTGAAGTCACAATCCTCTGGT |
| Human-CCL3-F | AGTTCTCTGCATCACTTGCTG |
| Human-CCL3-R | CGGCTTCGCTTGGTTAGGAA |
| Human-CCL5-F | CCAGCAGTCGTCTTTGTCAC |
| Human-CCL5-R | CTCTGGGTTGGCACACACTT |
| Human-CCL20-F | TGCTGTACCAAGAGTTTGCTC |
| Human-CCL20-R | CGCACACAGACAACTTTTTCTTT |
| Human-CXCL10-F | GTGGCATTCAAGGAGTACCTC |
| Human-CXCL10-R | TGATGGCCTTCGATTCTGGATT |
| Human-CXCL12-F | ATTCTCAACACTCCAAACTGTGC |
| Human-CXCL12-R | ACTTTAGCTTCGGGTCAATGC |
| Human-CXCL13-F | GCTTGAGGTGTAGATGTGTCC |
| Human-CXCL13-R | CCCACGGGGCAAGATTTGAA |
| Mouse-Gapdh-F | AGGTCGGTGTGAACGGATTTG |
| Mouse-Gapdh-R | TGTAGACCATGTAGTTGAGGTCA |
| Mouse-Becn1-F | ATGGAGGGGTCTAAGGCGTC |
| Mouse-Becn1-R | TCCTCTCCTGAGTTAGCCTCT |
| Mouse-Atg5-F | TGTGCTTCGAGATGTGTGGTT |
| Mouse-Atg5-R | GTCAAATAGCTGACTCTTGGCAA |
| Mouse-Atg10-F | GTAGTTACCAAGTGCCGGTTC |
| Mouse-Atg10-R | AGCTAACGGTCTCCCATCTAAA |
| Mouse-Atg12-F | TCCCCGGAACGAGGAACTC |
| Mouse-Atg12-R | TTCGCTCCACAGCCCATTTC |
| Mouse-Il6-F | CTGCAAGAGACTTCCATCCAG |
| Mouse-Il6-R | AGTGGTATAGACAGGTCTGTTGG |
| Mouse-Tnf-α-F | CAGGCGGTGCCTATGTCTC |
| Mouse-Tnf-α-R | CGATCACCCCGAAGTTCAGTAG |
| Mouse-Tlr2-F | CTCTTCAGCAAACGCTGTTCT |
| Mouse-Tlr2-R | GGCGTCTCCCTCTATTGTATTG |
| Mouse-Cxcl1-F | CTGGGATTCACCTCAAGAACATC |
| Mouse-Cxcl1-R | CAGGGTCAAGGCAAGCCTC |
| Mouse-Cxcl15-F | CAAGGCTGGTCCATGCTCC |
| Mouse-Cxcl15-R | TGCTATCACTTCCTTTCTGTTGC |
| Mouse-Cd68-F | TGTCTGATCTTGCTAGGACCG |
| Mouse-Cd68-R | GAGAGTAACGGCCTTTTTGTGA |
| Mouse-Itgam-F | ATGGACGCTGATGGCAATACC |
| Mouse-Itgam-R | TCCCCATTCACGTCTCCCA |
| Mouse-Cma1-F | TGGAGGCACGGAGTGCATA |
| Mouse-Cma1-R | AGGAGGACTGTTATAGACCTTCC |
| Mouse-Tpsab1-F | GCCAATGACACCTACTGGATG |
| Mouse-Tpsab1-R | GAGCTGTACTCTGACCTTGTTG |
| Mouse-Stat1-F | TCACAGTGGTTCGAGCTTCAG |
| Mouse-Stat1-R | GCAAACGAGACATCATAGGCA |
| Mouse-Stat3-F | CAATACCATTGACCTGCCGAT |
| Mouse-Stat3-R | GAGCGACTCAAACTGCCCT |
| Mouse-Il17-F | TTTAACTCCCTTGGCGCAAAA |
| Mouse-Il17-R | CTTTCCCTCCGCATTGACAC |
